# The influence of designer identity on design majors’ intention to use AIGC tools: an SEM-fsQCA analysis in China

**DOI:** 10.3389/fpsyg.2026.1908995

**Published:** 2026-08-24

**Authors:** Yaosheng Zhang, Yiping Luo

**Affiliations:** Art Design Department, Shangyu College, Shaoxing University, Shaoxing, China

**Keywords:** AIGC tools, design majors, designer identity, innovativeness, intention to use, perceived ease of use, self-efficacy

## Abstract

**Background:**

As AIGC is increasingly integrated into design education and professional practice, understanding the factors that drive design majors’ intention to use these tools has become an important issue. Existing studies have primarily explained technology adoption from cognitive and utilitarian perspectives, while paying limited attention to the role of designer identity.

**Methods:**

Drawing on Social Identity Theory (SIT), Innovation Diffusion Theory (IDT), and Innovation Resistance Theory (IRT), this study proposes an integrated theoretical model to examine how designer identity influences design majors’ intention to use AIGC tools. Survey data were collected from 615 design majors in China and analyzed using structural equation modeling (SEM) and fuzzy-set qualitative comparative analysis (fsQCA).

**Results:**

The results show that designer identity positively predicts intention to use AIGC tools both directly and indirectly through innovativeness and self-efficacy. In addition, perceived ease of use negatively moderates the relationship between designer identity and the intention to use AIGC tools, suggesting that highly accessible tools may sometimes raise concerns about professional distinctiveness among design majors with a strong designer identity. The fsQCA results further reveal three pathways associated with high intention to use AIGC tools: the Identity-Driven Innovation Pattern, the Identity-Driven Exploration Pattern, and the Tool-Empowered Pattern.

**Conclusion:**

This study contributes to the literature by introducing a professional identity-based lens to AIGC tool adoption research and demonstrating how designer identity, innovativeness, self-efficacy, and perceived ease of use jointly shape design majors’ engagement with emerging design technologies. The findings also provide practical implications for design education seeking to balance technological competence with the development of designer identity.

## 1 Introduction

The rapid proliferation of Artificial Intelligence Generated Content (AIGC) tools is fundamentally transforming creative practices in the design field (J. Wu et al., 2025). These tools can automatically generate multimodal content—including text, images, and layouts—thereby substantially improving design efficiency and lowering entry barriers (Park, 2026; Zhu and Zhang, 2025). In response, design education has begun to incorporate AIGC technologies into teaching practice (Huang et al., 2025), while the industry increasingly emphasizes designers’ ability to collaborate effectively with such tools (Wang and Chen, 2024a). Against this background, design majors’ intention to use AIGC tools has become a critical issue that affects not only their professional adaptability but also the broader intelligent transformation of the design industry.

Although the practical value of AIGC tools is widely acknowledged, design majors differ markedly in their intention to use these tools. Some design majors actively embrace AIGC to enhance their skills, whereas others remain hesitant or even resistant, fearing that it may undermine the professional value of designers (Huh et al., 2025; Wang, 2025). Existing studies have primarily explained such differences from cognitive and utilitarian perspectives, focusing on factors such as perceived usefulness, perceived ease of use (Li, 2025), and technology anxiety (Wang and Chen, 2024a). Although these factors are useful for explaining individuals’ rational evaluations of new technologies, they provide only a partial account of why design majors with similar evaluations of the same AIGC tool may still exhibit markedly different adoption intentions.

One possible explanation is that AIGC tools introduce a specific identity-related tension for design majors. This tension suggests that adopting these technologies is no longer merely a utilitarian choice but a decision deeply intertwined with one’s professional identity. As design majors undergo professional socialization, they gradually internalize the values, norms, and expectations associated with the design profession, including creativity, authorship, craftsmanship, and professional distinctiveness (Cavalcante, 2023; Huang et al., 2025; Kunrath et al., 2020). Unlike many other digital tools that primarily support designers’ work, AIGC tools increasingly participate in core creative activities such as idea generation, decision-making, and conceptual exploration (Quiñones-Gómez, 2026). Consequently, AIGC may blur the boundaries between human creativity and machine-generated content, thereby destabilizing established notions of authorship, problematizing the criteria for authentic design expertise, and potentially undermining the symbolic capital of professional competence (Zhang et al., 2025). As a result, design majors may perceive AIGC not only as a resource that enhances creative performance but also as a potential challenge to the autonomy and distinctiveness associated with the designer role. Their responses may therefore depend not only on instrumental evaluations of the technology but also on whether its use is perceived as compatible with their understanding of what it means to be a designer. Designer identity thus emerges as a key explanatory lens for heterogeneous acceptance patterns among design majors.

In this study, designer identity captures the degree to which design majors perceive being a designer as an important part of who they are and feel a sense of belonging to the designer community (Tajfel et al., 2001; Zhang and Zhang, 2016). This study provides a profession-specific lens for understanding how design majors interpret the implications of AIGC for their role, values, and future development as designers. Accordingly, design majors with stronger designer identity may evaluate AIGC not only based on its functional utility but also on whether its use is perceived as compatible with their identity as designers. Therefore, designer identity is proposed as the antecedent of this study.

Social Identity Theory (SIT) provides an appropriate starting point for understanding this phenomenon because it explains how individuals’ attitudes and behaviors are shaped by their identification with a social group and the values associated with that group (Hogg and Terry, 2000; Tajfel, 1972). For design majors, identifying strongly as designers encourages them to internalize professional norms that emphasize innovativeness, self-efficacy, continuous learning, and technology adoption. However, SIT primarily explains why professional identity matters; it fails to adequately elucidate how designer identity drives innovativeness and translates into technology adoption behaviors, nor can it account for why tool characteristics such as perceived ease of use may nevertheless trigger resistance when they threaten professional distinctiveness.

To address this limitation, this study integrates Social Identity Theory (SIT), Innovation Diffusion Theory (IDT), and Innovation Resistance Theory (IRT) into a unified theoretical framework. These theories address distinct yet complementary facets of AIGC adoption. First, SIT explains why designer identity becomes a fundamental psychological driver of technology-related attitudes by emphasizing the influence of membership in professional groups (Hogg and Terry, 2000; Tajfel, 1972). Second, IDT complements SIT by explaining how individual innovativeness and confidence in engaging with emerging technologies facilitate adoption (Rogers et al., 2014; Sahin, 2006). Specifically, design majors with a stronger designer identity are expected to exhibit greater innovativeness and confidence when learning emerging technologies, thereby increasing their intention to use AIGC tools. Third, IRT complements these positive mechanisms by explaining why favorable technological characteristics may not always encourage adoption (Leong et al., 2021; Ram and Sheth, 1989). For design majors with a strong designer identity, highly accessible AIGC tools may reduce the perceived exclusivity of professional design skills, thereby triggering identity-related resistance even when the technology is easy to use. Integrating these three theories therefore provides a more comprehensive explanation of AIGC adoption by simultaneously capturing identity formation, innovation acceptance, and innovation resistance.

Despite the growing body of research on the adoption of AIGC tools, several important gaps remain. First, prior research has focused on general cognitive and utilitarian factors such as technological characteristics, perceived usefulness, self-efficacy, and social influence (Li X. et al., 2025; Li Y. et al., 2025; Shimin et al., 2024; Wang, 2025), but pays less attention to designer identity as a core driver. Second, existing literature tends to apply SIT, IDT, and IRT in silos (Kuchmaner, 2026; Leong et al., 2021; Wang and Lin, 2019). There is a paucity of research integrating these lenses to explain how identity, innovativeness, and resistance jointly influence design majors’ intention to use AIGC tools. Third, no consensus has been reached regarding the effect of perceived ease of use on the intention to use AIGC tools (Fan and Jiang, 2024; Jiao and Cao, 2024; Shimin et al., 2024; Zhao et al., 2025), suggesting that its mechanism may depend on specific boundary conditions. Finally, most existing studies employ Structural Equation Modeling (SEM) to examine linear relationships (Li X. et al., 2025; Li Y. et al., 2025), while paying relatively little attention to the multiple configurational pathways through which different psychological conditions jointly produce high intention to use AIGC tools.

Consequently, this study adopts a novel perspective centered on designer identity. Drawing on Social Identity Theory, Innovation Diffusion Theory, and Innovation Resistance Theory, we construct an integrated theoretical model. In this model, designer identity serves as the independent variable; innovativeness and self-efficacy act as mediating variables; perceived ease of use functions as a moderating variable; and intention to use AIGC tools is the dependent variable. Using Structural Equation Modeling (SEM) and fuzzy-set Qualitative Comparative Analysis (fsQCA), we analyze survey data from 615 Chinese design majors to address three research questions: (1) How does designer identity influence design majors’ intention to use AIGC tools? (2) What role does perceived ease of use play in the theoretical model? (3) Which configurations of antecedent variables lead to high intention to use AIGC tools?

The main contributions and novelties of the present study are as follows:

(1) The integration of SIT, IDT, and IRT in the context of AIGC tools adoption in design education. This study integrates these three perspectives to explain how designer identity influences design majors’ intention to use AIGC tools through innovativeness and self-efficacy, while perceived ease of use serves as a contextual condition that may activate resistance related to professional identity. This framework offers a more context-sensitive explanation of AIGC tools adoption among design majors.
(2) The demonstration of the relationship between design majors’ designer identity and intention to use AIGC tools. This study introduces designer identity as an important explanatory lens for understanding AIGC tool adoption among design majors. Empirical results reveal that designer identity positively drives intention to use AIGC tools, with innovativeness and self-efficacy serving as key mediators, thereby highlighting the importance of cultivating designer identity and creative confidence alongside technical skills.
(3) New insights for understanding the role of perceived ease of use. This study advances TAM and IRT by demonstrating that perceived ease of use acts as a boundary condition, weakening the positive effect of designer identity on the intention to use AIGC tools.
(4) The identification of three configurations that lead to a high intention to use AIGC tools. This configurational approach reveals the complex interplay among designer identity, innovativeness, self-efficacy, and perceived ease of use, while providing a useful basis for developing differentiated educational strategies.

## 2 Literature review

### 2.1 Intention to use AIGC tools

Researchers have made substantial progress in exploring the factors, theoretical foundations, and methodological approaches associated with the intention to use AIGC tools. In terms of influencing factors, existing studies have identified personal factors, technological characteristics, costs, social influence, industry environment, task characteristics, and other determinants (Lai et al., 2025; Li X. et al., 2025; Li Y. et al., 2025; Shimin et al., 2024; Wang, 2025; Wang and Chen, 2024b; Zhu et al., 2025). Among these, personal factors have received particular attention, including cognitive factors such as perceived usefulness, perceived ease of use, and self-efficacy (Shimin et al., 2024; Wang, 2025); affective factors such as technology anxiety (Wang and Chen, 2024a), perceived risk (Li, 2025), optimism (Yao et al., 2025), and satisfaction (Fan and Jiang, 2024); and demographic factors such as gender (Tao et al., 2023) and educational level (Wang, 2025). Taken together, these studies show that individuals are generally more willing to adopt AIGC tools when they perceive it as useful, manageable, reliable, socially supported, and compatible with their work or learning tasks, yet often neglect the role of group-specific psychological variables, particularly innovativeness and professional identity, which are critical for understanding adoption in specialized fields like design.

Commonly used theoretical frameworks in this area are TAM, TPB, UTAUT, and AIDUA. For example, Li, using an extended UTAUT model, showed that effort expectancy, facilitating conditions, and perceived risk jointly shape designers’ intention to use AIGC tools (Li, 2025). In another study, Shimin and colleagues expanded the traditional TAM model by incorporating four additional factors—resource quality, self-efficacy, subjective norms, and convenience—to examine the intention to adopt AIGC technology in the Yixing Zisha design industry (Shimin et al., 2024). As research in this area advances, it has become common practice to merge classical theories, which improves how well these models explain actual user behavior. For instance, Wang and Chen (2024b), building on TAM and TPB, examined how perceived characteristics, risk, and trust influence designers’ adoption of AIGC tools. Using the AIDUA and TTF models, Lai et al. (2025) examined the impact of hedonic motivation, task–technology fit, effort expectancy, and emotions on designers’ acceptance of AIGC tools. These theoretical extensions have improved the explanatory power of conventional adoption models by recognizing that AIGC use depends on more than just technological functionality.

Nevertheless, the dominant logic of this research remains cognitive and utilitarian. Even when affective or social variables are included, adoption is usually conceptualized as an individual evaluation of whether a technology enhances performance, requires manageable effort, fits a task, or is supported by the surrounding environment. This approach is useful for explaining general technology acceptance, but it is less capable of accounting for profession-specific meanings attached to AIGC tools use. In design education, AIGC tools does not merely support peripheral administrative or technical activities. It increasingly participates in ideation, visualization, stylistic exploration, decision-making, and content production, all of which have traditionally been associated with designers’ creative judgment and professional expertise.

This distinction is theoretically important because design is both an instrumental activity and a symbolic professional practice. Evidence suggests that performance expectancy, effort expectancy, and social influence positively affect Chinese design students’ behavioral intention to use AIGC tools (Wang et al., 2025). For design students, the decision to use AIGC is not determined solely by whether the technology improves efficiency or performance. Design is also a professional and symbolic practice through which individuals demonstrate originality, authorship, and distinctive ways of thinking (Baboolall and Arora, 2026; Shahr et al., 2019; Zhang et al., 2025). Therefore, adopting AIGC may affect not only how design majors perform design tasks but also how they understand themselves as designers. A tool that expands creative exploration may be interpreted as supporting professional development, whereas the same tool may be viewed as outsourcing creative judgment, weakening personal authorship, bypassing skill formation, or reducing the distinction between trained designers and non-designers. General adoption variables alone cannot fully explain why design majors who perceive similar levels of usefulness or ease of use may nevertheless display different levels of hesitation or resistance.

Methodologically, researchers commonly employ SEM (Wang and Chen, 2024b), Partial Least Squares Structural Equation Modeling (PLS-SEM) (Li, 2025), semi-structured interviews combined with SEM (Zhu et al., 2025), and factor and weight analyses (Jiang et al., 2024). However, the predominant reliance on SEM has meant that prior research has focused mainly on the linear effects of individual variables, while paying less attention to the multiple configurational pathways through which different psychological conditions jointly produce a high intention to use AIGC tools.

In summary, prior studies have established the importance of functional beliefs, personal capabilities, emotional responses, social influence, and task characteristics in AIGC adoption. What remains underdeveloped is an explanation of how professional self-understanding shapes the meaning assigned to these technologies. To address this limitation, the present study moves beyond treating adoption solely as a cognitive-utilitarian evaluation and introduces designer identity as a profession-specific explanatory lens. This perspective provides the conceptual basis for examining how designer identity promotes design majors’ intention to use AIGC tools through innovativeness and self-efficacy, while also allowing perceived ease of use to operate as an identity-relevant boundary condition. The study further combines SEM and fsQCA to examine both linear relationships and alternative configurations associated with high intention to use AIGC tools.

### 2.2 Designer identity

Research on designer identity can be traced back to identity theory in sociology. In symbolic interactionism, George Mead argued that the construction of self-identity stems from receiving and interpreting feedback from others, through which individuals continuously shape and adjust their sense of self (Mead, 2015). Erikson, by contrast, emphasized that identity formation is a dynamic process of ongoing negotiation and adjustment between the individual self and the social environment (Erikson, 1968). These perspectives suggest that identity is not merely an internal psychological state, but a socially constructed understanding of who individuals are and who they aspire to become.

Drawing on Social Identity Theory, designer identity refers to the extent to which individuals identify with the designer community and attach emotional and evaluative significance to that membership (Tajfel et al., 2001; Zhang and Zhang, 2016). Specifically, designer identity can be understood as comprising three interrelated components (Ellemers et al., 1999). The cognitive component concerns self-categorization as a designer and the inclusion of the designer community. The affective component concerns feelings of belonging, attachment, and pride associated with being a designer or future designer. The evaluative component concerns the positive or negative value connotations attached to this group membership.

These components describe the structure of designer identity, but they do not fully specify its profession-specific meaning. The meaning attached to designer identity is shaped by the values, norms, and role expectations associated with design practice. Building on existing accounts of design practice, this study proposes creativity, authorship, craftsmanship, and professional distinctiveness as salient professional values through which individuals may understand what it means to be a designer and assess whether particular practices are consistent with the designer role (Cavalcante, 2023; Cross, 1982; Huang et al., 2025; Kunrath et al., 2020). These values are therefore treated not as separate dimensions of designer identity, but as interpretive resources that give profession-specific meaning to that identity while shaping design majors’ understanding of what it means to become a designer.

Creativity concerns the expectation that designers generate original ideas and solutions (Cross, 1982; Dorst and Cross, 2001; Kunrath et al., 2020), while authorship concerns whether design outcomes reflect the designer’s own judgment and professional voice (Cavalcante, 2023; Kolko, 2011; Sennett, 2008). Craftsmanship emphasizes the committed capacity to perform work well for its own sake through sustained practice and skill development (Cavalcante, 2023; Kolko, 2011; Sennett, 2008), whereas professional distinctiveness refers to the qualities that differentiate trained designers from non-designers (Kunrath et al., 2020; Tajfel et al., 2001).

AIGC may both reinforce and challenge these profession-specific meanings. On the one hand, it may expand design majors’ creative capabilities, support exploration and visualization, and enable new forms of human–AI collaboration. When perceived as a tool for creative enhancement, adoption of AIGC tools may strengthen design majors’ designer identity by supporting innovation and professional development. On the other hand, AIGC may be perceived as reducing personal contribution, weakening authorship, bypassing skill development, or blurring the boundary between trained designers and non-designers. Under such conditions, design majors with strong designer identity may experience uncertainty or resistance when AIGC is perceived as replacing rather than augmenting core design practices.

Accordingly, designer identity may produce both acceptance-oriented and resistance-oriented responses to the same technology. Design majors with stronger designer identity may be more motivated to engage with AIGC tools when adoption is understood as experimentation, professional advancement, and participation in the future of design. The same identity may also heighten sensitivity to technologies perceived as weakening creativity, authorship, craftsmanship, or professional distinctiveness. This dual possibility is especially relevant for design majors because their professional identities are still developing. Design education involves not only learning software, production techniques, and problem-solving methods, but also internalizing professional values, standards, and role expectations. Through studio practice, critique, project experience, and interaction with teachers and peers, design majors gradually form beliefs about what designers should be capable of and what kind of designers they wish to become.

In summary, the present study focuses on the acceptance-oriented mechanism by proposing that designer identity strengthens innovativeness and self-efficacy. Design majors who strongly identify with the designer role may be more inclined to value experimentation with emerging methods and to invest effort in mastering tools relevant to future professional practice. Innovativeness and self-efficacy may therefore transmit the positive influence of designer identity to intention to use AIGC tools. At the same time, ease of use may have an identity-sensitive effect. When high accessibility is interpreted as reducing the exclusivity or symbolic value of professional design skills, perceived ease of use may weaken, rather than strengthen, the positive relationship between design majors’ designer identity and their intention to use AIGC tools. These arguments establish the conceptual basis for the mediation and moderation hypotheses developed in the following sections.

## 3 Theoretical foundation

### 3.1 Social identity theory

Social identity theory (SIT) provides the principal theoretical foundation for explaining how designer identity influences design majors’ intention to use AIGC tools. Since its introduction by Tajfel in the 1970s, SIT has become one of the most influential theories for understanding how group membership shapes individuals’ attitudes and behaviors (Hogg, 2016). Social identity refers to the part of an individual’s self-concept derived from membership in a social group, together with the value and emotional significance attached to that membership (Tajfel, 1972). According to SIT, social identity develops through three related processes: social categorization, social comparison, and social identification (Lange et al., 2012). Social categorization is the process by which individuals are clustered into groups; social comparison is the process by which characteristic group features are interpreted and valued; social identification speaks to one key reason why groups of people differ from object categories (Lange et al., 2012).

In the context of this study, SIT helps explain how designer identity may influence design majors’ intention to use AIGC tools. Through social categorization, design majors who strongly identify themselves as future designers are more likely to adopt the values and expectations associated with the design profession. Through social comparison, they tend to view creativity and innovation as important characteristics that distinguish designers from other groups. As a result, a stronger designer identity may encourage higher levels of innovativeness, which in turn increases design majors’ willingness to experiment with and adopt AIGC tools. At the same time, identifying with the designer role can strengthen design majors’ confidence in their ability to learn and apply new technologies. This greater sense of self-efficacy may further promote their intention to use AIGC tools. Therefore, SIT offers a useful perspective on how designer identity influences AIGC adoption through innovativeness and self-efficacy. However, SIT alone does not explain how this identity translates into technology adoption behaviors, which motivates the incorporation of Innovation Diffusion Theory.

### 3.2 Innovation diffusion theory

Innovation Diffusion Theory (IDT) was first proposed by Rogers in 1962 and further refined by him in 1995. This theory aims to describe how members of society adopt novel products, practices, or ideas (Rogers et al., 2014). The diffusion of innovation process comprises four main elements: innovation, communication channels, time, and social system (Sahin, 2006). The innovation decision process involves five stages: knowledge, persuasion, decision, implementation, and confirmation (Sahin, 2006).

Notably, the applicability of IDT in studying individual usage intentions has been validated across multiple research contexts. For instance, Wang and Lin (2019) integrated the TTF model with IDT theory to analyze young users’ intention to use mobile cloud healthcare systems; Agag and El-Masry (2016) combined the TAM model with IDT and introduced trust influence factors to explore the mechanisms affecting consumers’ willingness to purchase travel products online; another study integrated the UTAUT2 model, ECT model and IDT to analyze academic users’ continuous behavior intention of Mobile Visual Search (Wu and Zhang, 2023).

In this research context, IDT exhibits a dual connection with the research theme. Primarily, as an innovative technology in the design industry, the diffusion process of AIGC tools among design majors aligns with IDT theory’s core logic of innovation propagation within social systems, providing a theoretical foundation for the pathway “Innovativeness → intention to use AIGC tools.” Accordingly, IDT complements SIT by explaining how designer identity may foster innovativeness and self-efficacy, thereby facilitating the adoption of AIGC tools. Nevertheless, positive perceptions of innovation do not always lead to adoption intention. Under certain circumstances, the same technological characteristics may also evoke resistance, which is addressed by Innovation Resistance Theory.

### 3.3 Innovation resistance theory

The Innovation Resistance Theory (IRT), introduced by Sheth, employs a negative perspective to examine why individuals reject certain innovations. It identifies Habit and Risk as the primary factors driving resistance to innovation. Innovation resistance comprises two categories: functional barriers and psychological barriers. Functional barriers encompass product usage patterns, perceived value, and usage risks, while psychological barriers stem from traditions and norms of the customers and perceived product image (Ram and Sheth, 1989). Subsequent studies have consistently validated the effectiveness of this theory. For instance, Sadiq et al. (2021) found that all five barriers significantly hindered consumers’ purchase of eco-friendly cosmetics. Leong et al. (2021) conducted a meta-analysis of 26 studies and found that these five barrier factors effectively explain consumer resistance to innovation.

Against the backdrop of transitioning to digital design, despite the increasing accessibility of AIGC tools, many design majors remain hesitant or even resistant to them (Huh et al., 2025; C. Wang, 2025). This practical contradiction has prompted academia to shift its research focus toward the mechanism through which perceived ease of use influences the intention to use AIGC tools. Existing research on the impact of “perceived ease of use” on the intention to use AIGC tools remains inconclusive: some studies suggest a positive driving effect, while others indicate it may trigger professional identity anxiety, thereby inhibiting adoption intention (Fan and Jiang, 2024; Shimin et al., 2024). This divergence suggests that perceived ease of use may moderate the mechanism underlying intention to use AIGC tools. Therefore, this study proposes to explore the influence of designer identity on the intention to use AIGC tools, using IRT, and to examine whether perceived ease of use moderates this relationship. This aims to deepen our understanding of the complex attitudinal mechanisms toward AIGC tools within this group.

Taken together, SIT explains why designer identity matters, IDT explains how it promotes innovation adoption through innovativeness and self-efficacy, and IRT explains why perceived ease of use may simultaneously trigger resistance when AIGC threatens profession-specific values. Together, these theories provide a coherent framework for understanding both the enabling and constraining mechanisms underlying design majors’ intention to use AIGC tools.

## 4 Research hypotheses

### 4.1 Designer identity and intention to use AIGC tools

According to SIT, individuals who identify with a specific social group (e.g., “designers”) internalize that group’s norms and behavioral patterns (Tajfel et al., 2001). The design field is actively embracing AIGC as an innovation tool (Li X. et al., 2025; Li Y. et al., 2025). Consequently, design majors who identify as “designers” are more likely to perceive the use of AIGC tools as consistent with their professional identity and a means to enhance their competitiveness, thereby generating a stronger intention to use such tools. The theory further suggests that individuals tend to adopt behaviors consistent with their reference group to maintain a positive self-concept (Hogg and Terry, 2000). When AIGC tools come to be regarded within design communities as a marker of professional competence or an essential skill, design majors with a heightened sense of group identification are more likely to embrace them—both to avoid being left behind and to affirm their position as insiders. Therefore, we propose the following hypothesis:

*H1*: Designer identity positively influences design majors’ intention to use AIGC tools.

### 4.2 Designer identity and innovativeness

Innovativeness (INN) is an individual trait reflecting people’s inclination to become technological pioneers and thought leaders (Parasuraman and Colby, 2007). Innovativeness also involves an individual’s intention to try innovations (Agarwal and Prasad, 1998; Jackson et al., 2013). According to Hernández et al. (2018), innovation is a core value of the design profession. For design majors, strong identification with the designer role implies a deeper internalization of the expectation of being innovative. This identity motivates individuals to seek new methods and technologies (such as AIGC tools) to express their professional uniqueness, leading to higher levels of personal innovativeness (Tajfel et al., 2001). Moreover, the essence of the design profession lies in solving problems through innovation (Dorst, 2011). Taken together, design majors with a strong designer identity are more likely to deeply internalize these innovation-centered behavioral norms (Tajfel et al., 2001), thereby effectively stimulating innovativeness.

*H2*: Designer identity positively influences design majors’ innovativeness.

### 4.3 Innovativeness and intention to use AIGC tools

According to IDT and related research, an individual’s innovativeness is a key predictor of their intention to adopt new technologies (Agarwal and Karahanna, 2000; Rogers et al., 2014). Karahanna’s research further confirmed that individuals with stronger innovativeness are more willing to adopt new technologies (Karahanna et al., 1999). Accordingly, design majors with stronger innovativeness are more inclined to explore and use new technologies, such as AIGC tools, that offer innovative possibilities.

*H3*: Innovativeness positively influences design majors’ intention to use AIGC tools.

### 4.4 The mediating role of innovativeness

Integrating the logical chains of H2 and H3, designer identity fosters design majors’ internalization of innovation-centered professional norms, thereby enhancing their innovativeness. When design majors’ innovativeness is strengthened, they exhibit stronger intention to explore and adopt AIGC tools. Thus, Innovativeness mediates the relationship between designer identity and design majors’ intention to use AIGC tools. Therefore, we propose the hypothesis:

*H4*: Innovativeness mediates the relationship between design majors’ designer identity and their intention to use AIGC tools.

### 4.5 Designer identity and self-efficacy

Self-efficacy refers to an individual’s belief about their capabilities to execute behaviors necessary to produce specific performance attainments (Bandura and Wessels, 1997). An individual’s self-efficacy is not static but is profoundly influenced by their professional community (Bandura and Wessels, 1997). Firstly, the vicarious experiences provided by social models impact an individual’s self-efficacy. Design majors with high designer identity are more inclined to internalize the designer community as a significant reference group. This strong sense of belonging and identification prompts them to observe the behaviors, values, and achievements of other designers (Tajfel et al., 2001). Witnessing the success of their designer models strongly suggests that observers themselves possess the potential for success. Secondly, social persuasion can also enhance an individual’s sense of self-efficacy. Design majors with a strong designer identity are more receptive to encouragement and support from the design community, internalizing it as a source of self-efficacy. Furthermore, accumulating successful experiences is a crucial source of self-efficacy. For example, Chien et al. (2022) surveyed 285 industrial-design students and found that those with more maker-space experience exhibited significantly stronger self-efficacy in problem solving, information collecting, and model prototyping than their less experienced peers. Therefore, we propose the following hypothesis:

*H5*: Designer identity positively influences design majors’ self-efficacy.

### 4.6 Self-efficacy and intention to use AIGC tools

Existing studies have repeatedly shown that self-efficacy plays a meaningful role in shaping people’s intention to adopt artificial intelligence technologies (Falebita and Kok, 2025; Liu et al., 2025). Work in specific areas, such as fashion design, further supports this view by revealing a clear positive link between users’ self-efficacy and their acceptance of AIGC tools (Yang and Jin, 2025). This influence seems to work in more than one way. Some findings indicate that self-efficacy can indirectly shape adoption intentions through factors such as perceived ease of use or AI attitude (Li X. et al., 2025; Li Y. et al., 2025; Zhao et al., 2025). For design majors—who regularly face the challenge of learning new creative tools and methods—having a strong sense of self-efficacy matters a lot. It boosts their belief that they can handle emerging technologies, including AIGC tools. Based on this reasoning, we expect that design majors with higher self-efficacy will show a stronger intention to use these tools. Accordingly, we put forward the following hypothesis:

*H6*: Self-efficacy positively influences design majors’ intention to use AIGC tools.

### 4.7 The mediating role of self-efficacy

Integrating the logical chains of H5 and H6, we propose that self-efficacy mediates the relationship between designer identity and the intention to use AIGC tools. Specifically, designer identity promotes self-efficacy among design majors through three pathways: vicarious experiences from social models, social persuasions, and accumulated successful experiences. Enhanced self-efficacy, in turn, increases their intention to use AIGC tools. Therefore, we propose the following hypothesis:

*H7*: Self-efficacy mediates the relationship between design majors’ designer identity and their intention to use AIGC tools.

### 4.8 Innovativeness and self-efficacy

According to IDT theory and Karahanna’s research findings, individuals with stronger innovative capabilities are more willing to embrace new technologies (Karahanna et al., 1999; Rogers et al., 2014) and are motivated to invest time in experimenting with emerging technologies such as AIGC. Continuous experimentation with new technologies yields multiple outcomes, such as mastering tool functions, becoming familiar with design processes, and solving typical problems. These experiences serve as one source of self-efficacy (Chien et al., 2022). Furthermore, Fredrickson’s research indicates that innovation is often accompanied by positive emotions (Fredrickson, 2001), and positive emotions can enhance self-efficacy (Bandura and Wessels, 1997). Therefore, it is reasonable to infer that innovativeness can promote an individual’s technological adaptation and innovation capabilities, generating positive emotions that enhance their self-efficacy. Therefore, we propose the hypothesis:

*H8*: Innovativeness positively influences design majors’ self-efficacy.

### 4.9 The serial mediating roles of innovativeness and self-efficacy

The relationships proposed above suggest that innovativeness and self-efficacy may play the serial mediating roles between designer identity and intention to use AIGC tools. First, strong designer identity encourages design majors to internalize creativity, experimentation, and technological adaptability as professional values. This identity-based motivation strengthens innovativeness and increases the intention to use AIGC tools. Second, exploration provides design majors with opportunities to acquire knowledge, master tool functions, solve operational problems, and accumulate successful experiences. These experiences strengthen self-efficacy by providing evidence that design majors can effectively use emerging design technologies. Third, greater self-efficacy increases intention to use AIGC tools because confident design majors are more likely to expect that they can control the technology, overcome difficulties, and integrate it into design practice. Therefore, we propose the following hypothesis:

*H9*: Innovativeness and self-efficacy serially mediate the relationship between design majors’ designer identity and their intention to use AIGC tools.

### 4.10 The moderating role of perceived ease of use

Perceived ease of use (PEOU) refers to a user’s belief that a system is easy to use and free of effort (Davis, 1989). According to IRT, designers’ adherence to traditional design paradigms constitutes a source of resistance to the adoption of new technologies (Ram and Sheth, 1989). AIGC tools are simple to operate and substantially lower the barrier to entry for design and creative work, thereby disrupting traditional models of creative labor (Şahin and Karayel, 2024). Design skills that traditionally required years of training can now be approximated through simple text prompts. For design majors with a strong designer identity, this may raise concerns about the dilution of unique skills and professional expertise, potentially leading to resistance to AIGC tools. Moreover, limited attention to designer identity may prevent researchers from fully understanding design work. For instance, the abandonment of certain design practices may not be driven by instrumental concerns such as efficiency, but rather by attempts to avoid perceived threats to designer identity (Plattner et al., 2012). Therefore, when AIGC tools are perceived as ease of use, individuals with a strong designer identity may experience greater identity threat, which, in turn, may reduce their intention to use AIGC tools. Thus, we propose the following hypothesis:

*H10*: Perceived ease of use negatively moderates the effect of design majors’ designer identity on their intention to use AIGC tools.

### 4.11 Research aim, hypothesis summary, and conceptual model

**Figure 1:**
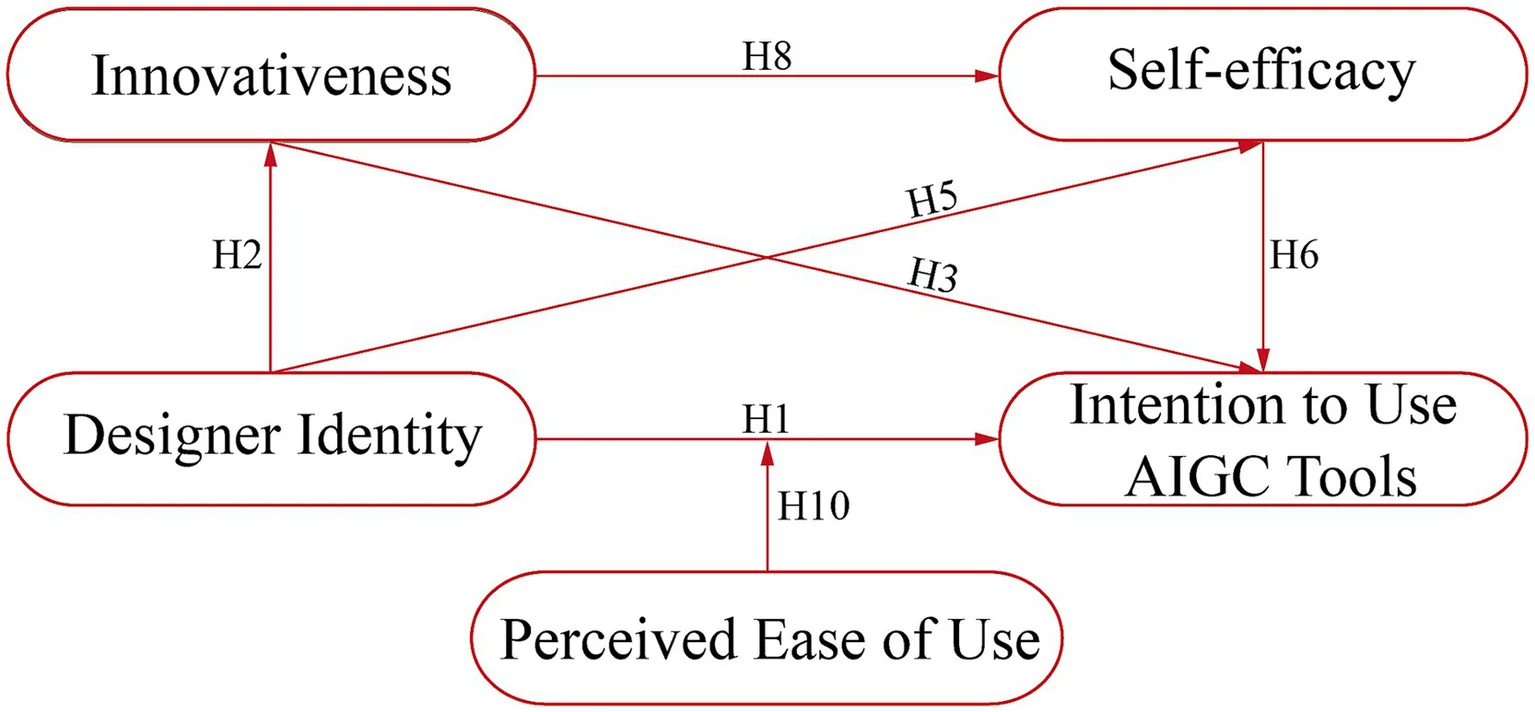
Conceptual model.

The aim of this study is to explain how designer identity shapes design majors’ intention to use AIGC tools. Specifically, the study examines the direct effect of designer identity; the serial mediating effects of innovativeness and self-efficacy; and the moderating effect of perceived ease of use. In addition to examining these linear relationships, the study uses fsQCA to identify alternative configurations that lead to a high intention to use AIGC tools. The preceding subsections developed ten hypotheses based on SIT, IDT, and IRT. The proposed hypotheses are summarized in Table 1. While Figure 1 presents the conceptual model.

**Table 1:** Summary of the proposed hypotheses.

Hypotheses	Proposed relationship	Effect type
H1	Designer identity positively influences design majors’ intention to use AIGC tools.	Direct
H2	Designer identity positively influences design majors’ innovativeness.	Direct
H3	Innovativeness positively influences design majors’ intention to use AIGC tools.	Direct
H4	Innovativeness mediates the relationship between design majors’ designer identity and their intention to use AIGC tools.	Mediating
H5	Designer identity positively influences design majors’ self-efficacy.	Direct
H6	Self-efficacy positively influences design majors’ intention to use AIGC tools.	Direct
H7	Self-efficacy mediates the relationship between design majors’ designer identity and their intention to use AIGC tools.	Mediating
H8	Innovativeness positively influences design majors’ self-efficacy.	Direct
H9	Innovativeness and self-efficacy serially mediate the relationship between design majors’ designer identity and their intention to use AIGC tools.	Serial Mediating
H10	Perceived ease of use negatively moderates the relationship between design majors’ designer identity and their intention to use AIGC tools.	Moderating

## 5 Methodology

### 5.1 Instruments and measurements

To test the proposed research model, we developed a survey instrument covering five latent constructs. Designer identity served as the independent variable, while innovativeness and self-efficacy were treated as mediating variables. Intention to use AIGC tools functioned as the dependent variable, and perceived ease of use was included as a moderating variable. All measurement items were drawn or adapted from previous scales. Responses were collected using a seven-point Likert format, ranging from 1 (“strongly disagree”) to 7 (“strongly agree”). To ensure validity, the scale was revised in three stages. First, a panel of eight university faculty experts reviewed and refined the items. Second, a pre-survey with 50 participants was conducted to screen items with factor loadings below 0.6. Finally, the scale was further polished and adjusted. The final main scale comprised 23 items (see Table 2). Four demographic variables—gender, major, educational background, and grade level—were also included to describe sample characteristics. In addition, one AIGC tool recognition item was designed for precise respondent screening, and two lie-detection items were embedded to ensure data reliability.

**Table 2:** The information of the measurement model.

Construct	Items	Loading	Cronbach’s *α*	CR	AVE
Designer Identity (DI)	When the outside world offers positive recognition of designers, I feel a sense of belonging and pride.	0.754	0.842	0.844	0.520
	When the outside world criticizes designers, I feel criticized too.	0.696			
	When discussing designers, I typically use “we.”	0.753			
	I’m pleased when others see me as a designer.	0.738			
	If negative reports about designers appear in the media, I take it as a warning to myself.	0.661			
Innovativeness (INN)	People often seek my advice on new technologies.	0.774	0.890	0.892	0.624
	Within my social circle, I’m usually the first to adopt new technologies.	0.811			
	I typically explore and grasp cutting-edge tech products and services independently.	0.823			
	Within my areas of interest, I stay abreast of the latest technological developments.	0.806			
	Compared to others, I encounter fewer issues when utilizing new technological tools.	0.732			
Self-efficacy (SE)	I am confident in my ability to proficiently use design software to complete my assignments.	0.706	0.852	0.855	0.541
	I believe I can quickly learn and master new design tools or techniques.	0.751			
	I believe I possess creativity and can propose innovative design solutions.	0.741			
	I can effectively communicate my design intent through visual and verbal means.	0.766			
	I believe I can maintain high productivity under pressure and complete design tasks on schedule.	0.713			
Perceived ease of use (PEOU)	I find the AIGC tool’s interface clear and intuitive, allowing me to quickly locate the features I need.	0.769	0.829	0.829	0.550
	I believe that even without prior experience, I could master the basic operations of the AIGC tool through simple experimentation.	0.639			
	I find the AIGC tool’s usage prompts and feedback messages explicit, eliminating any confusion during operation.	0.759			
	I find the AIGC tool’s operational steps concise and straightforward.	0.790			
Intention to use AIGC tools (ITU)	I think using AIGC tools is a good idea.	0.831	0.889	0.890	0.669
	I believe I am highly likely to use AIGC tools.	0.810			
	I plan to use AIGC tools in the future.	0.833			
	I am willing to use AIGC tools.	0.797			

### 5.2 Sampling and data collection

This study employed snowball sampling, a non-probability sampling technique. With assistance from design faculty members and class advisers at multiple universities across China, questionnaires were distributed to students in design-related disciplines, including visual communication design, art design, packaging design, product design, digital media art design, environmental design, textile art design, industrial design, and character design. To improve participant engagement, each valid response that passed verification was compensated with 3 yuan. Data were collected from January 15 to March 13, 2026, yielding 948 completed questionnaires. During data cleaning, responses were excluded if participants had no prior exposure to AIGC tools, failed both lie-detection items, or completed the questionnaire in less than 60 seconds. After screening, 615 valid responses remained, including 370 undergraduate students (60.16%) and 245 vocational college students (39.84%).

### 5.3 Data analysis

Data were analyzed using SPSS 27.0, AMOS 26.0, and fsQCA 4.0. The analytical procedures were conducted in six stages.

First, common method bias was assessed using a single-factor confirmatory factor analysis following the recommendations of Podsakoff et al. (2003) and Harman (1976). Second, the reliability and validity of the measurement model were evaluated using Cronbach’s *α*, exploratory factor analysis (EFA), confirmatory factor analysis (CFA), composite reliability (CR), and average variance extracted (AVE). The assessment criteria followed commonly adopted methodological guidelines for multivariate analysis and structural equation modeling (Fornell and Larcker, 1981; Hair et al., 2019; Hu and Bentler, 1999; Kline, 2023). Third, structural equation modeling (SEM) was performed using SPSS 27.0 to estimate the direct relationships among designer identity, innovativeness, self-efficacy, and intention to use AIGC tools (Hair et al., 2019). Fourth, we examined the indirect and serial mediating effects of innovativeness and self-efficacy using percentile bootstrapping with 5,000 resamples and 95% confidence intervals (Hayes, 2017). Fifth, hierarchical regression analysis was conducted using SPSS 27.0 to test whether perceived ease of use moderated the relationship between designer identity and intention to use AIGC tools. The predictor variables were mean-centered before the interaction term was calculated to reduce potential multicollinearity (Cohen et al., 2013). Finally, fsQCA 4.0 was used as a complementary configurational approach to identify alternative combinations of conditions associated with high intention to use AIGC tools. Following the principles of set-theoretic analysis (Pappas and Woodside, 2021; Ragin, 2009), the procedure included data calibration, necessity analysis, truth-table construction, and sufficiency analysis. This approach complements the symmetric linear-effect analyses by examining how different combinations of designer identity, innovativeness, self-efficacy, and perceived ease of use may jointly lead to high intention to use AIGC tools.

## 6 Results

### 6.1 Common method bias

Common method bias was statistically assessed following the recommendations of Podsakoff et al. (2003) and Harman (1976). Specifically, a single-factor confirmatory factor analysis was conducted by loading all measurement items onto a single latent factor. As shown in Table 3, the single-factor model exhibited a poor fit. Specifically, the RMSEA was significantly higher than 0.05, CMIN/DF substantially exceeded 3, and the CFI, GFI, NFI, RFI, IFI, and TLI were all below 0.9. Taken together, these results suggest that no single factor accounted for the covariance among the observed variables, indicating that common method bias was unlikely to be a serious concern in this study.

**Table 3:** Results of the common method bias testing.

Fit indices	RMSEA	CMIN/DF	CFI	GFI	NFI	RFI	IFI	TLI
Value	0.120	9.898	0.742	0.682	0.722	0.694	0.743	0.717

### 6.2 Reliability and validity testing

The reliability and validity of the measurement model were evaluated in four stages.

First, the internal consistency analysis was conducted using SPSS 27.0. As shown in Table 2, Cronbach’s α values for the five constructs ranged from 0.829 to 0.890, exceeding the recommended threshold of 0.70, and the overall Cronbach’s α was 0.941. These results indicate satisfactory internal consistency reliability for all constructs (Hair et al., 2019).

Second, EFA was conducted using SPSS 27.0. The Kaiser–Meyer–Olkin (KMO) value was 0.952, well above the threshold of 0.7. The Bartlett’s test of sphericity was significant (*p* < 0.001), indicating that the sample data were suitable for factor analysis (Hair et al., 2019). Factor analysis of the 23 items yielded five factors that jointly explained 67.85% of the cumulative variance. Varimax rotation was used to simplify the factor structure. After seven iterations, the rotation converged, and all items loaded on the five prespecified factors as expected. This result indicates that the factor structure had high stability and explanatory power.

Third, CFA was conducted using AMOS 26.0 to evaluate the measurement model. The model exhibited a good fit to the data: RMSEA = 0.045, CMIN/DF = 2.247, GFI = 0.932, NFI = 0.940, IFI = 0.966, CFI = 0.966, and TLI = 0.960. Collectively, these indices indicate that the hypothesized five-factor measurement model provided a satisfactory representation of the observed data (Fornell and Larcker, 1981; Hair et al., 2019; Hu and Bentler, 1999).

Fourth, construct reliability and validity were assessed. Standardized factor loadings ranged from 0.639 to 0.833. The CR value for each construct exceeded 0.70, indicating satisfactory construct reliability, and the AVE values for each construct exceeded 0.50, providing support for convergent validity (Fornell and Larcker, 1981; Hair et al., 2019). Discriminant validity was further evaluated by comparing the proposed five-factor model with several alternative measurement models. As reported in Table 4, the five-factor model exhibited the best overall fit and significantly outperformed the competing models. These findings support the empirical distinctiveness of designer identity, innovativeness, self-efficacy, perceived ease of use, and intention to use AIGC tools (Cheung et al., 2024). Overall, the measurement model demonstrated satisfactory reliability, convergent validity, and discriminant validity.

**Table 4:** Discriminant validity test results.

Model	*χ* ^2^	*df*	χ^2^/*df*	NFI	CFI	RMSEA
Original five-factor model	494.373	220	2.247	0.940	0.966	0.045
Four-factor model 1(a + e,b,c,d)	1145.703	224	5.115	0.860	0.884	0.082
Four-factor model 2(a + b,c,d,e)	1167.800	224	5.213	0.858	0.881	0.083
Three-factor model 1(a + b + c,d,e)	1413.244	227	6.226	0.828	0.850	0.092
Three-factor model 2(a + b,c + d,e)	1414.557	227	6.231	0.827	0.851	0.092
Two-factor model 1(a + b + c,d + e)	1683.249	229	7.350	0.795	0.817	0.102
Two-factor model 2(a + b,c + d + e)	1914.481	229	8.360	0.766	0.788	0.110
Single-factor model (a + b + c + d + e)	2276.616	230	9.898	0.722	0.742	0.120

a, designer identity; b, innovativeness; c, self-efficacy; d, perceived ease of use; e, intention to use AIGC tools.

### 6.3 Model fit

Following commonly adopted SEM guidelines (Hair et al., 2019; Kline, 2023), the structural equation model demonstrated an acceptable overall model fit. RMSEA = 0.0497, CMIN/DF = 2.520, GFI = 0.939, NFI = 0.944, IFI = 0.966, and CFI = 0.966. The RMSEA was below 0.05, CMIN/DF was below 3, and all reported incremental fit indices exceeded 0.90. Taken together, these results indicate that the proposed structural model provided an acceptable representation of the observed data and was suitable for subsequent hypothesis testing.

### 6.4 Direct effect

The results of the direct effect tests are presented in Table 5. Designer identity positively influenced intention to use AIGC tools (*β* = 0.264, *p* < 0.001), innovativeness (*β* = 0.480, *p* < 0.001), and self-efficacy (*β* = 0.305, *p* < 0.001). Innovativeness positively influenced self-efficacy (*β* = 0.516, *p* < 0.001) and intention to use AIGC tools (*β* = 0.238, *p* < 0.001). Self-efficacy also positively influenced intention to use AIGC tools (*β* = 0.218, *p* < 0.001). Therefore, hypotheses H1, H2, H3, H5, H6, and H8 were supported.

**Table 5:** Results of direct effect testing.

Model	Regression equation		Model fit indices			Standardized effect and significance	
	Outcome variable	Predictor variable	*F*	*R*	*R* ^2^	*β*	t
1	Intention to use AIGC tools	Designer identity	203.208	0.499	0.249	0.499	14.255***
2	Innovativeness	Designer identity	183.706	0.480	0.231	0.480	13.554***
3	Self-efficacy	Designer identity	318.808	0.714	0.510	0.305	9.471***
		Innovativeness				0.516	15.985***
4	Intention to use AIGC tools	Designer identity	117.845	0.605	0.367	0.264	6.725***
		Innovativeness				0.238	5.445***
		Self-efficacy				0.218	4.731***

***Indicates *p* < 0.001.

### 6.5 Indirect effect

The results of the indirect effect tests are presented in Table 6. Innovativeness (*β* = 0.114, 95% CI does not include 0) and self-efficacy (*β* = 0.066, 95% CI does not include 0) significantly mediated the relationship between designer identity and intention to use AIGC tools; Innovativeness and self-efficacy (*β* = 0.054, 95% CI does not include 0) exhibited a significant serial mediation effect between designer identity and intention to use AIGC tools. Therefore, hypotheses H4, H7, and H9 were supported.

**Table 6:** Results of indirect effect testing.

Hypotheses	Paths	*β*	Boot SE	95%CI	Results
H4	Mediating path 1: DI → INN→ITU	0.114	0.023	[0.071, 0.164]	Supported
H7	Mediating path 2: DI → SE → ITU	0.066	0.022	[0.026, 0.115]	Supported
H9	Serial mediating path: DI → INN→SE → ITU	0.054	0.016	[0.024, 0.087]	Supported

### 6.6 Moderating effect

**Figure 2:**
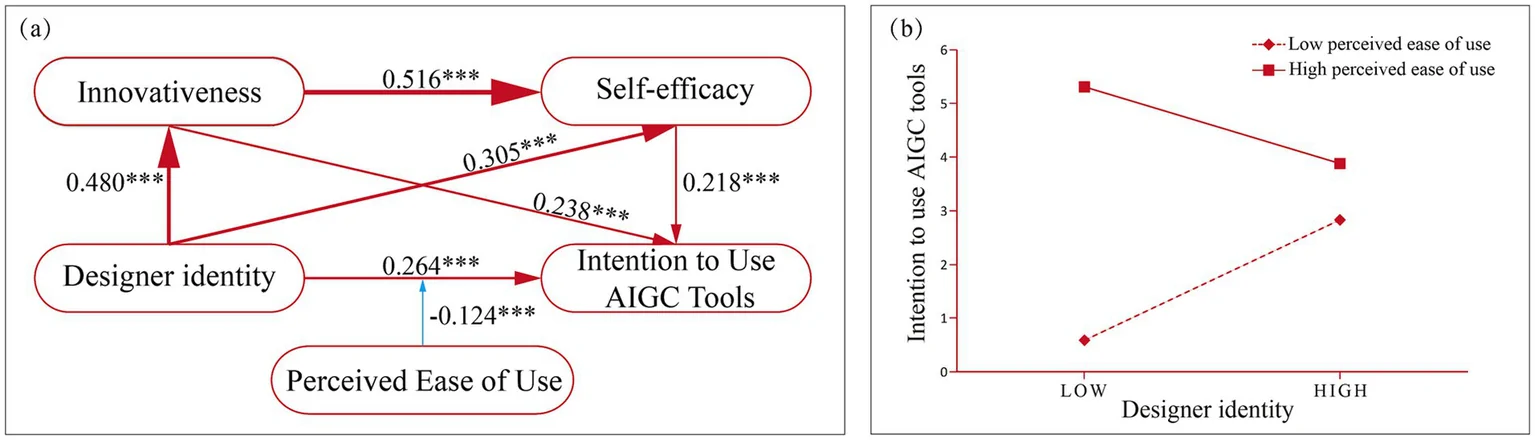
Results of the conceptual model. **(a)** Path coefficients of the conceptual model; **(b)** moderation effect results of perceived ease of use.

Hierarchical linear regression was used to examine the moderating role of perceived ease of use in the relationship between designer identity and intention to use AIGC tools (Cohen et al., 2013). First, designer identity and perceived ease of use were mean-centered. Second, the interaction term between designer identity and perceived ease of use was calculated. Finally, a hierarchical linear regression analysis was conducted to test the moderating effect of perceived ease of use, with results summarized in Table 7. The analysis revealed the interaction term had a negative effect on the intention to use AIGC tools (*β* = −0.124, *p* < 0.001, 95% CI = [−0.245, −0.092]). As illustrated in Figure 2B, perceived ease of use negatively moderated the relationship between designer identity and intention to use AIGC tools, supporting hypothesis H10. Specifically, the positive association between designer identity and intention to use AIGC tools became weaker as perceived ease of use increased. Additionally, the path coefficients and significance levels for the moderated serial mediation model are shown in Figure 2A.

**Table 7:** Results of moderating effect testing.

Model	Regression equation		Model Fit Indices				Standardized effect and significance	
	Outcome variable	Predictor variable	ΔF	*R*	*R* ^2^	Δ*R*^2^	*β*	*t*
1	ITU	DI	203.208	0.499	0.249	0.248	0.499	14.255***
5		DI	273.597	0.694	0.481	0.479	0.185	5.336***
		PEOU					0.575	16.541***
9		DI	18.602	0.705	0.496	0.494	0.175	5.090***
		PEOU					0.577	16.828***
		DI × PEOU					−0.124	−4.313***

***Indicates *p* < 0.001.

### 6.7 Fuzzy-set qualitative comparative analysis

Fuzzy-set qualitative comparative analysis (fsQCA) was employed to examine how different combinations of antecedent conditions were associated with high intention to use AIGC tools. FsQCA complements the SEM results by identifying multiple configurations that lead to the same outcome, thereby providing additional insights beyond the linear effects revealed by SEM (Fiss, 2011; Pappas and Woodside, 2021; Ragin, 2009).

#### 6.7.1 Variable selection and data calibration

First, based on the preceding theoretical analysis and empirical evidence, this study adopted designer identity, innovativeness, self-efficacy, and perceived ease of use as antecedent variables for the fSQCA analysis, with intention to use AIGC tools serving as the outcome variable.

Next, the mean value for each antecedent variable was calculated. The calibration anchors for each variable were then determined using 0.95 for “fully in,” 0.5 for “intermediate,” and 0.05 for “fully out” (Pappas and Woodside, 2021; Ragin, 2009). Data calibration was subsequently performed with the calibrate function in fsQCA 4.0.

Finally, following Cangialosi (2023), any fuzzy scores that were exactly 0.5 during calibration were uniformly adjusted to 0.501. The data calibration results are presented in Table 8.

**Table 8:** Calibration anchors for each condition.

Variable	Calibrated anchors		
	Fully in	Intermediate	Fully out
DI	6.600	5.600	4.000
INN	6.400	4.800	3.400
SE	6.600	5.400	4.000
PEOU	6.575	5.500	4.000
ITU	6.750	5.750	4.000

#### 6.7.2 Analysis of necessary conditions

Following Ragin (2009), a consistency threshold of 0.90 was adopted to identify necessary conditions. As shown in Table 9, the consistency levels for all conditions are below 0.9, indicating that none of the conditions is necessary for the outcome. Individual conditions alone do not provide sufficient explanatory power for the outcome. Thus, intention to use AIGC tools can be understood as being driven by multiple antecedent factors.

**Table 9:** Necessary condition analysis results.

Conditional variable	ITU		~ITU	
	Consistency	Coverage	Consistency	Coverage
DI	0.773484	0.808499	0.534326	0.481194
~DI	0.503663	0.556615	0.787353	0.749669
INN	0.769245	0.799660	0.516235	0.462353
~INN	0.482800	0.536687	0.776311	0.743490
SE	0.787290	0.816172	0.519422	0.463931
~SE	0.482901	0.538383	0.794182	0.762850
PEOU	0.786672	0.859421	0.474690	0.446795
~PEOU	0.493622	0.521684	0.850642	0.774543

#### 6.7.3 Configurations for high intention to use AIGC tools

Following established fsQCA guidelines (Fiss, 2011; Pappas and Woodside, 2021; Ragin, 2009), this study applied fsQCA 4.0 to examine the causal configurations. During construction of the truth table, the consistency threshold was set to 0.8, the minimum acceptable number of cases was set to 11, and the PRI value was maintained above 0.68 using natural breakpoints. In this study, conditions that appear in both the intermediate and parsimonious solutions were considered as core conditions, while those appearing only in the intermediate solution were classified as peripheral conditions.

The configuration analysis results are shown in Table 10. Four antecedent configurations for high intention to use AIGC tools were obtained: S1, S2, S3a, and S3b. Their consistency coefficients were 0.916, 0.929, 0.918, and 0.917, respectively, with an overall consistency of 0.881. These results indicate that the four configurations are sufficient conditions for high intention to use AIGC tools. The overall coverage indicates that these configurations explain 74.4% of cases with high intention to use AIGC tools. Based on the research context and the distribution of core conditions across configurations, this study classifies the antecedent configurations into three patterns. Configuration S1, centered on designer identity, innovativeness, and self-efficacy, is defined as the Identity-Driven Innovation Pattern. Configuration S2, centered on designer identity, innovativeness, and perceived ease of use, is defined as the Identity-Driven Exploration Pattern. Configurations S3a and S3b, centered on self-efficacy and perceived ease of use, are grouped as the Tool-Empowered Pattern.

**Table 10:** Configurations lead to high intention to use AIGC tools.

Conditional variable	High levels of ITU			
	S1	S2	S3a	S3b
DI	●	●	●	
INN	●	●		●
SE	●		●	●
PEOU		●	●	●
Raw coverage	0.608839	0.601126	0.620198	0.636802
Unique coverage	0.034540	0.026827	0.045899	0.062503
Consistency	0.916410	0.929134	0.918210	0.917184
Solution coverage	0.744067			
Solution consistency	0.880854			

Black circles (●) indicate the presence of a condition. Large circles represent core conditions, and small circles denote peripheral conditions. A blank space indicates a “do not care” condition.

Identity-Driven Innovation Pattern. Configuration S1 indicates that designer identity, innovativeness, and self-efficacy are core conditions that lead to high intention to use AIGC tools. This configuration is substantively consistent with the key conditions involved in the serial mediation pathway proposed in H9, although it should not be interpreted as direct evidence of mediation. Rather, it provides configurational support for the underlying motivational mechanism: a strong designer identity may motivate design majors to engage with AIGC tools, innovativeness leads them to regard these tools as essential for realizing creative ideas, and self-efficacy provides the confidence needed to use them. Consequently, this pattern underscores the dominant role of designer identity in the adoption process.

Identity-driven exploration pattern. Configuration S2 shows that a high intention to use AIGC tools can be achieved when designer identity, innovativeness, and perceived ease of use as core conditions. This pattern suggests that for design majors with strong designer identity and innovativeness, perceived ease of use lowers adoption barriers and reduces technology-related anxiety, thereby strengthening their intention to use AIGC tools. It also reveals a key distinction from S1: whereas S1 suggests that strong design identity can drive adoption regardless of usability, S2 highlights the catalytic role of tool usability. When design majors perceive AIGC tools as easy to use, this perception further activates usage intention already rooted in professional identity and innovative needs. Thus, this pattern highlights the enabling role of technological characteristics in the adoption process.

Tool-empowered pattern. Configurations S3a and S3b show that high intention to use AIGC tools can be fully achieved when self-efficacy and perceived ease of use are present as core conditions. Designer identity or innovativeness can act as supplementary factors. This means that the main driving force of tool-enabled empowerment lies in the interaction between self-efficacy and perceived ease of use. These reduce psychological barriers and operational costs for AIGC tool users. In configuration S3a, designer identity is a supplementary condition. This shows that strengthening designer identity can enhance intention to use AIGC tools. Moreover, even when design majors’ designer identity is not pronounced, high self-efficacy and perceived ease of use are still sufficient to generate strong usage intention. Similarly, in configuration S3b, innovativeness is the supplementary condition. This suggests that if tool usability is sufficient and self-efficacy is high, a moderate level of innovation can strengthen intention to use AIGC tools.

#### 6.7.4 Assessing the robustness of fsQCA results

The robustness of fsQCA configuration analysis results can be established if the subset relationships remain stable under parameter adjustments (Skaaning, 2011). This study verified the reliability of the results through three sensitivity tests. First, increasing the frequency threshold from 11 to 12 did not alter the configurations. Second, lowering the PRI consistency threshold from 0.68 to 0.6 also yielded identical configurations. Finally, during calibration, setting the “fully in” and “fully out” anchors at 0.85 and 0.15, respectively, while keeping the intermediate point unchanged, resulted in no substantial changes to the configuration structure upon re-analysis. The above test results indicate that the high intention to use AIGC tools identified in this study are highly robust.

## 7 Discussion

The aim of this study was to explain how designer identity is associated with design majors’ intention to use AIGC tools by integrating insights from SIT, IDT, and IRT within a professional identity perspective. The SEM results supported all ten hypotheses. Specifically, designer identity is related to intention both directly and indirectly through innovativeness and self-efficacy, while perceived ease of use weakens this positive relationship. The fsQCA analysis further reveals multiple configurations associated with high intention to use AIGC tools. The following sections discuss these findings in relation to prior research and consider their theoretical and practical implications.

### 7.1 A context-specific integration of SIT, IDT, and IRT for explaining AIGC tools adoption

This study integrates SIT, IDT, and IRT to propose an integrative theoretical framework for explaining design majors’ intention to use AIGC tools in the context of design education. By combining these three theories, the framework addresses certain limitations of theoretical frameworks such as TAM and UTAUT—particularly their limited attention to professional identity—and provides a complementary perspective on technology acceptance behaviors where professional identity plays a salient role.

With SIT as the logical starting point, the framework places designer identity at the core of the model. The findings show that strong designer identity not only directly promotes intention to use AIGC tools but also stimulates innovativeness within the IDT framework and enhances self-efficacy through the internalization of group norms. In doing so, this study extends technology acceptance research beyond instrumental rationality to the socio-emotional dimension of identity.

By bridging IDT and SIT, this study reveals a dual-mediation mechanism involving innovativeness and self-efficacy. Designer identity enhances the intention to use AIGC tools by strengthening innovativeness and self-efficacy, indicating that design majors’ intention is shaped not only by tool attributes but also by their pursuit of innovation and confidence in their own abilities (Wang et al., 2025), both of which are activated by designer identity.

Most notably, the introduction of IRT specifies the contextual role of perceived ease of use. The results show that perceived ease of use negatively moderates the relationship between designer identity and their intention to use AIGC tools. Specifically, for design majors with strong designer identity, highly user-friendly AIGC tools may actually inhibit adoption, creating a usability paradox. This finding suggests that the role of perceived ease of use may be more context-dependent than is typically assumed in traditional TAM-based research (Jiao and Cao, 2024; Shimin et al., 2024). In particular, the positive implications of ease of use may become less pronounced when it interacts with strong designer identity, potentially because highly accessible tools carry identity-relevant meanings for some design majors.

### 7.2 The relationship between designer identity and intention to use AIGC tools

This study introduces designer identity as an important explanatory lens for understanding AIGC tools adoption among design majors. The empirical results support the hypothesis that designer identity positively influences intention to use AIGC tools, both directly and indirectly. This finding is consistent with Social Identity Theory, which argues that individuals tend to engage in behaviors that reinforce their group membership and maintain a positive social identity (Hogg, 2016; Tajfel et al., 2001). As AIGC increasingly becomes part of professional design practice, design majors with stronger designer identity may perceive mastering AIGC tools as consistent with becoming competent future designers.

First, the direct-effect results indicate that designer identity has a significant positive effect on the intention to use AIGC tools, supporting the SIT view that individuals strengthen their sense of group belonging by adopting group-relevant technologies. Compared with studies grounded in technology acceptance models such as TAM, UTAUT, and UTAUT2—such as those by Shimin et al. (2024), Wang and Chen (2024b), Li (2025)—this study highlights an important difference in explanatory focus. Prior studies mainly emphasized cognitive and utilitarian factors, whereas the present study clarifies the independent role of designer identity as an affective driver. It therefore demonstrates the direct motivational value of designer identity in shaping intention to use AIGC tools and addresses the missing identity dimension in technology acceptance research.

Second, this study identifies a psychological transmission chain that influences the intention to use AIGC tools. The mediation results show that innovativeness and self-efficacy function as both separate mediators and sequential mediators in the relationship between designer identity and intention to use AIGC tools. On the one hand, these findings are consistent with Bandura’s account of the sources of self-efficacy. On the other hand, unlike studies by Wang et al. (2024), Shimin et al. (2024), and Li (2025), which focused on general motivational variables, this study treats innovativeness—one of the core characteristics of the designer group—as a mediating variable. In this way, it reveals the mediating role of group-specific psychological variables in technology adoption. This finding provides additional evidence for the role of group-specific psychological factors in technology adoption and offers a more nuanced understanding of AIGC acceptance among design majors.

### 7.3 New insights for understanding the role of perceived ease of use

One of the most novel findings of this study is that perceived ease of use negatively moderates the relationship between designer identity and intention to use AIGC tools. Contrary to the assumptions of traditional TAM research (Davis, 1989), the positive influence of designer identity becomes weaker as AIGC tools are perceived as easier to use. This finding deserves particular attention because it challenges the widely accepted assumption that greater usability invariably promotes technology adoption.

A possible explanation can be found by integrating SIT with IRT. From the perspective of TAM, higher perceived ease of use lowers learning costs and reduces the effort required to use new technologies, thereby encouraging adoption (Davis, 1989). However, IRT suggests that technological characteristics may simultaneously activate psychological barriers when they threaten users’ established norms and values (Leong et al., 2021; Ram and Sheth, 1989). In the design context, these norms are closely associated with creativity, authorship, craftsmanship, and professional distinctiveness, all of which constitute important foundations of designer identity (Cavalcante, 2023; Kunrath et al., 2020).

For design majors with a strong designer identity, therefore, the high accessibility of AIGC tools may carry an unintended symbolic meaning. When sophisticated design outputs can be generated easily, and without years of professional training, ease of use may reduce the perceived exclusivity of design expertise and blur the boundary between trained designers and non-designers. As a result, although perceived ease of use reduces the functional effort required to adopt AIGC, it may also heighten concerns that the technology devalues professional training, weakens authorship and craftsmanship, and undermines the distinctiveness of the designer role. This interpretation is consistent with recent studies showing that design majors are concerned that AI-generated design may diminish designers’ professional value and weaken human creativity (Huh et al., 2025; Wang, 2025). Thus, the same characteristic that facilitates technology acceptance at the functional level may generate resistance at the identity level, particularly among individuals whose self-concept is strongly tied to their professional identity as designers.

Importantly, this finding also helps reconcile inconsistent conclusions in previous AIGC research. While many studies have reported a significant positive relationship between perceived ease of use and intention to use AIGC tools (Shimin et al., 2024; Zhao et al., 2025), other studies have found negative effects (Fan and Jiang, 2024; Jiao and Cao, 2024). Our results suggest that these inconsistencies may arise because perceived ease of use functions as a context-dependent boundary condition rather than a universal antecedent. Specifically, when designer identity becomes highly salient, users evaluate ease of use not only in terms of efficiency but also in terms of its symbolic implications for professional identity. Under such circumstances, technological accessibility may simultaneously facilitate adoption and activate identity-related resistance. Therefore, rather than viewing perceived ease of use as an inherently positive or negative determinant of technology acceptance, this study suggests that its influence depends on users’ identity-based interpretations of technology. This finding extends the application of TAM and IRT by demonstrating that technological characteristics possess dual functions: they reduce functional barriers while simultaneously shaping users’ perceptions of professional identity and occupational distinctiveness. In creative disciplines such as design, the role of designer identity and its interpretive lens should therefore be considered alongside traditional cognitive evaluations when explaining AIGC adoption.

### 7.4 Three configurations leading to high intention to use AIGC tools

The fsQCA results indicate that three configurational patterns lead to a high intention to use AIGC tools. The core conditions of the Identity-Driven Innovation Pattern (S1) are designer identity, innovativeness, and self-efficacy; those of the Identity-Driven Exploration Pattern (S2) are designer identity, innovativeness, and perceived ease of use; and those of the Tool-Empowered Pattern (S3) are self-efficacy and perceived ease of use. Unlike prior studies that have largely focused on linear variable effects, this study uses fsQCA analysis to reveal four insights. First, designer identity plays a pivotal role. It serves as a core condition in S1 and S2 and as a supporting condition in S3a, indicating that it can both directly drive intention to use AIGC tools and enhance the effects of other variables. Second, innovativeness has flexible value. It can operate as a core driver in S1 and S2 and as a differentiating condition within the self-efficacy-dominated S3 pattern. Third, perceived ease of use has a dual role. It can catalyze professional value in S1 and S2, but may weaken perceived professional value in S3. The configurational findings further illustrate the context-dependent role of perceived ease of use. Its contribution to high adoption intention varies across combinations of identity, innovativeness, and self-efficacy. Although these findings do not directly test the interaction identified in the SEM analysis, they are consistent with the broader conclusion that perceived ease of use does not operate as a uniformly positive condition across all identity and psychological configurations. Fourth, S3 shows higher coverage than S1 and S2, especially S3b. One possible interpretation is that, for a substantial subset of respondents, tool-related confidence and usability may be sufficient for high adoption intention even when designer identity is not a core condition. Future research could examine whether this pattern varies across stages of designer identity development.

### 7.5 Practical implications

First, the SEM results suggest that designer identity positively influences design majors’ intention to use AIGC tools. This finding indicates that introducing AIGC into design education may be more acceptable when framed in relation to design majors’ understanding of professional design practice rather than presented solely as technical training. Accordingly, educators may consider incorporating limited and critically structured human–AIGC collaborative activities into existing design courses. For example, design majors could compare AIGC-generated proposals with human-developed alternatives, explain their revision decisions, and reflect on the roles of judgment, originality, contextual understanding, and aesthetic evaluation in the design process. Such activities may help design majors perceive AIGC use as potentially compatible with, rather than necessarily threatening to, their emerging designer identity.

Second, the mediating roles of innovativeness and self-efficacy suggest that design majors’ willingness to engage with AIGC may be supported by learning activities that provide opportunities for exploration and manageable mastery experiences. Educators may therefore introduce AIGC tasks progressively, beginning with relatively structured exercises before moving to more open-ended design problems. Optional experimentation tasks, peer demonstrations, reflective critiques, and examples of different AIGC-supported workflows may also be used to encourage exploration and reduce uncertainty.

Third, the negative moderating effect of perceived ease of use indicates that highly accessible AIGC tools may not be interpreted positively by all design majors. In particular, when design majors strongly identify with the designer role, very low entry barriers may raise concerns about the distinctiveness of professional expertise. Therefore, educators should avoid presenting ease of use as the sole educational value of AIGC. Teaching could instead emphasize that generating an output is different from producing a contextually appropriate and professionally defensible design solution. Classroom discussions may focus on evaluating generated content, identifying limitations, documenting decision processes, and explaining where professional judgment remains necessary.

Fourth, the fsQCA findings indicate that high intention to use AIGC tools can emerge through multiple configurations rather than a single universal pathway. For design majors represented by the Identity-Driven Innovation Pattern, instructional approaches may consider linking AIGC use to professional designer identity and providing opportunities for creative experimentation. For those represented by the Identity-Driven Exploration Pattern, reducing operational barriers through accessible tools may support engagement when accompanied by strong designer identity and innovativeness. For design majors represented by the Tool-Empowered Pattern, technical skill development should be accompanied by guidance that prevents the self-suggestion that tools should take precedence during technical learning. While design majors benefit from AIGC tools, they should also be guided toward a rational understanding of value creation in the design industry. Furthermore, learning activities that integrate professional knowledge with technology can help design majors recognize that the organic integration of AIGC tools and professional expertise is necessary to maximize value and, on this basis, gradually build a deeper sense of designer identity.

Overall, these findings suggest that design educators may benefit from considering different combinations of motivational and technological factors when introducing AIGC tools, rather than relying on a single instructional approach.

## 8 Conclusion

This study examined how designer identity influences design majors’ intention to use AIGC tools in the context of design education in China. Drawing on Social Identity Theory, Innovation Diffusion Theory, and Innovation Resistance Theory, the study developed an integrated theoretical model in which designer identity was positioned as the independent variable, innovativeness and self-efficacy as mediators, perceived ease of use as a moderator, and intention to use AIGC tools as the dependent variable. Based on survey data from 615 Chinese design majors, this study employed SEM and fsQCA to investigate the linear and configurational mechanisms underlying the intention to use AIGC tools.

The SEM findings show that designer identity significantly and positively influences intention to use AIGC tools. In addition, innovativeness and self-efficacy both play important mediating roles. Designer identity enhances design majors’ innovativeness and self-efficacy in using emerging technologies, which in turn strengthens their intention to use AIGC tools. The results also support a serial mediating effect (designer identity → innovativeness → self-efficacy → intention), consistent with H9, indicating that designer identity promotes intention to use AIGC tools through the serial pathway of innovativeness and self-efficacy.

Another important finding is that perceived ease of use negatively moderates the relationship between designer identity and intention to use AIGC tools. For design majors with strong designer identity, highly accessible AIGC tools may create concerns about the dilution of professional skills and creative value, thereby weakening their intention to use such tools. This finding extends traditional technology acceptance research by revealing the potential usability paradox of AIGC tools in design education.

The fsQCA results further demonstrate that high intention to use AIGC tools can be achieved through multiple pathways, including the Identity-Driven Innovation Pattern, the Identity-Driven Exploration Pattern, and the Tool-Empowered Pattern. These findings indicate that AIGC adoption among design majors is characterized by causal complexity and cannot be fully explained through a single linear relationship.

Theoretically, this study contributes to the literature by introducing a professional identity-based lens into AIGC adoption research and by integrating SIT, IDT, and IRT into a unified framework. Practically, the findings suggest that design education should move beyond purely technical training and place greater emphasis on cultivating designer identity, innovativeness, and self-efficacy in the era of human–AI collaboration.

## 9 Research limitations and future directions

First, the generalizability of the findings is limited by the sample characteristics. Although the sample size was sufficient for the purposes of the present analysis, the participants were relatively homogeneous in terms of educational background and cultural context. The study therefore does not fully account for possible differences among regions, institutional types, or study levels, such as distinctions between undergraduate and graduate design majors. As a result, the generalizability of the findings to wider student populations remains limited. Subsequent research should incorporate more diverse samples and conduct comparative studies across populations and cultural settings to further test the robustness and wider applicability of the findings.

Second, the model’s explanatory scope remains limited. The mediating mechanisms examined in this study were limited to innovativeness and self-efficacy. Other factors that may shape design majors’ intentions to use AIGC tools, such as AI literacy, instructor support, prior experience with digital technologies, and the perceived affordances of specific tools, were not incorporated into the model. This may have constrained the explanatory scope of the study. Subsequent research could develop more comprehensive conceptual models that include these additional variables, thereby delivering a more nuanced understanding of how design majors form intentions to adopt AIGC tools.

Third, the study is constrained by its reliance on self-reported cross-sectional data. Although single-factor test was conducted to measure potential common method bias, this procedure cannot entirely eliminate concerns regarding common method variance. Future studies should consider combining survey data with behavioral records, experimental assessments, interviews, or evaluations from educators and colleagues. Such triangulation would help strengthen the validity of the findings and provide a more grounded account of design majors’ actual engagement with AIGC tools.

## Data Availability

The raw data supporting the conclusions of this article will be made available by the authors, without undue reservation.
